# Effectiveness of Posterior Chain Resistance Exercises and Spinal Stabilization Exercises on Pain, Strength, and Disability in Runners With Non-specific Low Back Pain

**DOI:** 10.7759/cureus.114958

**Published:** 2026-08-21

**Authors:** Shantanu S Kshirsagar, Smita C Patil

**Affiliations:** 1 Department of Sports Physiotherapy, Krishna College of Physiotherapy, Krishna Vishwa Vidyapeeth (Deemed to be University), Karad, IND

**Keywords:** disability, muscle strength, pain, posterior chain resistance exercises, recreational runners, rehabilitation, spinal stabilization exercises

## Abstract

Background

Non-specific low back pain (NSLBP) is a common musculoskeletal condition among recreational runners and may negatively affect pain, trunk muscle strength, and functional ability. Repetitive mechanical loading during running can contribute to lumbopelvic dysfunction and reduced spinal stability. Although spinal stabilization exercises are widely used in rehabilitation, the additional benefits of posterior chain resistance exercises remain unclear.

Objectives

This study aimed to determine the effectiveness of posterior chain resistance exercises combined with spinal stabilization exercises on pain, strength, and disability in recreational runners with NSLBP and to compare their effects with spinal stabilization exercises alone.

Methods

Thirty-six male recreational runners aged 18-25 years with NSLBP were recruited and randomly allocated into two groups (n=18 each). Group A received posterior chain resistance exercises combined with spinal stabilization exercises, while Group B received spinal stabilization exercises alone. Interventions were administered every three days for six weeks. Pain was assessed using the Numeric Pain Rating Scale (NPRS), trunk muscle strength using a pressure biofeedback unit (PBU), and disability using the Modified Oswestry Disability Index (MODI). Outcome measures were recorded before and after the intervention period.

Results

Significant improvements in pain, strength, and disability were observed within both groups following six weeks of intervention (p≤0.001). In Group A, pain scores decreased from 6.22±0.73 to 2.72±0.66, strength improved from 41.88±1.72 to 43.88±2.15, and disability scores decreased from 22.44±2.70 to 19.22±3.07. In Group B, pain scores decreased from 6.11±0.75 to 3.27±0.75, strength improved from 41.83±2.01 to 42.94±2.18, and disability scores decreased from 22.00±2.35 to 18.88±2.49 (p≤0.001). Between-group analysis demonstrated significantly greater pain reduction in Group A compared with Group B (p=0.025). No significant differences were found between groups for strength (p=0.206) or disability (p=0.723).

Conclusion

Both interventions were effective in improving pain, strength, and disability in recreational runners with NSLBP. However, the addition of posterior chain resistance exercises resulted in greater pain reduction than spinal stabilization exercises alone, suggesting that it may be a valuable component of rehabilitation programs for recreational runners with NSLBP.

## Introduction

Low back pain (LBP), one of the most frequently reported symptoms in musculoskeletal conditions worldwide, represents a major reason for disability, reduced quality of life, and healthcare utilization across all age groups. It affects individuals regardless of occupation, lifestyle, or physical activity level and continues to pose a significant public health challenge globally [1]. The burden of recurrent and chronic LBP has increased significantly in recent years, leading to considerable socioeconomic consequences and loss of productivity [2].

Among the various forms of LBP, non-specific low back pain (NSLBP) represents the bulk of clinical presentations. NSLBP is characterized by pain and discomfort in the lumbar region without a clearly identifiable pathological cause such as fracture, infection, inflammatory disease, or nerve root compression [3]. The condition is multifactorial in nature and is influenced by biomechanical, neuromuscular, psychological, and lifestyle-related factors [4]. Since most cases do not have a proven structural pathology, conservative therapies are the main focus of therapeutic strategies in order to improve functional capacity and reduce symptoms [5].

Exercise therapy is widely recognized and proven effective as a non-pharmacological approach for the management of chronic NSLBP. Several studies have shown that structured exercise programs can reduce pain, improve function, and enhance quality of life in affected individuals [6]. The capacity of segmental stabilization exercises to target the deep trunk muscles in charge of preserving spinal stability during movement and functional activities has been highlighted [7].

Spinal stabilization exercises are designed to improve the activation and coordination of key stabilizing muscles, including the multifidus and transversus abdominis. Dysfunction of these muscles has been connected to a decrease in spinal stability and recurrent episodes of LBP [8]. According to clinical practice guidelines, exercise is advised as an intervention, including stabilization exercises. These are an essential component of the rehabilitation program for NSLBP [9]. Additionally, active rehabilitation techniques targeted at function restoration and handicap reduction are implemented in European recommendations for long-term NSLBP [10].

Recent research continues to support the effectiveness of exercise therapy in the management of persistent LBP. Exercise therapy significantly improves pain and physical function when compared to minimal intervention or standard care, according to systematic reviews [11]. These findings highlight the importance of identifying exercise interventions that not only enhance treatment outcomes but also target the underlying impairments associated with NSLBP.

LBP impacts a variety of individuals, such as both the general public and those who engage in regular physical activity, including recreational and competitive athletes. Even while running is frequently featured as a healthy kind of exercise due to its positive effects on cardiovascular fitness and overall health, runners are also exposed to repetitive mechanical loading and mechanical load on the musculoskeletal system [12]. Prolonged exposure to repetitive mechanical loads may increase the risk of developing or sustaining LBP when combined with inadequate muscular strength, poor movement control, or biomechanical dysfunction [13].

Current clinical guidelines emphasize the role of physical activity and self-management. Effective LBP is managed by encouraging active interventions aimed at improving physical performance while avoiding excessive reliance on passive treatments [14]. Contemporary evidence further supports individualized exercise programs that address specific deficits in strength, endurance, and motor control [15]. In this context, improving spinal stability and overall kinetic chain function has become an important focus of rehabilitation strategies for physically active populations.

Spinal stability has been widely investigated in rehabilitation research due to its critical role in maintaining optimal spinal function and preventing musculoskeletal disorders. Sannasi et al. described spinal stability as the capacity of the spine to maintain appropriate alignment and control under physiological loads through the coordinated interaction of passive structures, active muscular systems, and neural control mechanisms [16]. Dysfunction within any of these components, particularly the diaphragm and other key trunk stabilizing muscles, may compromise spinal stability and increase susceptibility to pain and injury. Consequently, rehabilitation programs targeting trunk stabilization have become a cornerstone of NSLBP management.

Evidence-based guidelines from the advance care planning recommend non-invasive treatments, particularly exercise therapy, as first-line management for chronic LBP [17]. Similarly, systematic reviews have shown that exercise interventions effectively reduce pain and disability. However, the effectiveness of treatment may vary depending on the exercise modality implemented [18]. This has led researchers to investigate whether combining stabilization exercises with other forms of strengthening may provide additional benefits.

Running-related LBP has gained increasing attention in the field of sports rehabilitation. A systematic review identified LBP as a common musculoskeletal complaint among both recreational and competitive runners, highlighting the need for targeted preventive and therapeutic interventions. Recreational runners often participate in training programs without structured strength conditioning, potentially increasing the risk of muscular imbalances and reduced lumbopelvic stability. Such deficits may contribute to altered movement patterns and elevated lumbar spine mechanical stress [19].

The World Health Organization (WHO) has recently emphasized the importance of non-surgical and non-pharmacological interventions, including organized exercise programs, in the management of persistent LBP [20]. These recommendations reinforce the growing recognition of exercise as a safe, accessible, and effective intervention for individuals with persistent LBP.

While spinal stabilization exercises have demonstrated effectiveness in improving pain and function, emerging rehabilitation approaches increasingly emphasize the role of the posterior chain. Weakness or dysfunction within these muscles may impair lumbopelvic control and increase mechanical stress on the lumbar region. Strengthening the posterior chain may therefore complement spinal stabilization by enhancing overall kinetic chain performance and reducing excessive loading on the spine.

Despite growing interest in posterior chain strengthening, evidence regarding the combined effectiveness of posterior chain strengthening and spinal stabilization exercises in recreational runners with NSLBP remains limited. Therefore, this research attempts to investigate the efficiency of posterior chain resistance exercises combined with spinal stabilization exercises on pain, strength, and disability within recreational runners with NSLBP. These results may enhance current rehabilitation frameworks by informing more effective treatment strategies for physically active individuals.

## Materials and methods

Study design and setting

This randomized research utilized a pre-test and post-test study design. The study was conducted at Chhatrapati Shivaji Stadium and various athletics academies in Karad, India, from November 2025 to May 2026. Participants were recruited through convenience sampling and were subsequently allocated into two groups using an odd-even random allocation method. Ethical approval was obtained from the Institutional Ethics Committee of Krishna Vishwa Vidyapeeth (Deemed to be University) prior to the commencement of the study (approval number: KVV/IEC/10/2025; date: 05/11/2025). All participants provided written informed consent before enrollment in the study.

Participants

Participants were identified and diagnosed in the hospital electronic medical record using ICD-10 code M54.5 (Low back pain, unspecified). The term "non-specific low back pain (NSLBP)" used throughout this manuscript refers to the clinical/research classification applied after excluding specific pathoanatomical causes of LBP, as detailed in the Inclusion and Exclusion Criteria below, rather than to a distinct ICD diagnostic code. The study included 36 male recreational runners between 18 and 25 years of age. Recreational runners were defined as individuals who participated in running as a leisure activity rather than as professional athletes. All participants who met the eligibility criteria provided written informed consent prior to enrollment in the trial. Participants were randomly allocated into two groups: Group A (experimental group; n=18), which received posterior chain resistance exercises combined with spinal stabilization exercises, and Group B (control group; n=18), which received spinal stabilization exercises only. The training program was organized progressively, beginning with diaphragmatic breathing and basic trunk activation to establish neuromuscular control, followed by more demanding stabilization and functional exercises. This progression was intended to gradually increase trunk muscle activation and load tolerance while maintaining appropriate movement control.

The sample size was calculated using the formula $n=\frac{2\left(Z_{\alpha/2}+Z_{\beta}\right)^2\sigma^2}{d^2}$ where n is the required sample size per group, Zα/2​ is the standard normal deviate corresponding to the desired level of significance (1.96 for a two-sided 5% significance level), Zβ​ is the standard normal deviate corresponding to the desired study power (0.84 for 80% power), σ is the estimated standard deviation of the primary outcome, and d is the expected difference in means between the two groups.

As shown in Table 1, both groups performed exercises three times per week for six weeks with progressive increases in intensity. The experimental group received posterior chain resistance exercises using elastic resistance bands along with progression in repetitions and hold durations. The control group performed spinal stabilization exercises with progressive increases in repetitions, sets, hold times, and exercise difficulty. The experimental group focused on posterior chain strengthening, whereas the control group emphasized spinal stability and flexibility.

**Table 1:** Exercise protocol for the experimental group (posterior chain resistance exercises with spinal stabilization) and control group (spinal stabilization exercises alone) over a six-week intervention period

Posterior chain resistance exercises (experimental group)		
Exercises	Doses (3 times/week)	
Week	0-3 weeks (red elastic resistance band)	4-6 weeks (blue elastic resistance band)
Resistance band	10 reps	15 reps
Oblique crunch (supine)		
Trunk rotation		
Trunk chop		
Trunk extension		
Diagonal patterns		
Thoracic elevation with hands straight at the front (4-6 weeks)	5 reps with 30 sec hold	5 reps with 45 sec hold
Dead lift (elastic band)	7 reps	14 reps
Spinal stabilization exercises (control group)		
Exercises	Doses (3 times/week)	
Week	0-3 weeks	4-6 weeks
Bridge	3 sets, 10 reps	3 sets, 10 reps
Bridge progression (a single-leg bridge exercise)		
Plank (supporting the lower body on the knees)	2 sets, 30 sec hold	2 sets, 60 sec hold
Plank progression (supporting the lower body on the feet)		
Side plank (supporting the lower body on the knees)	2 sets, 30 sec hold	2 sets, 60 sec hold
Side plank progression (supporting the lower body on the feet)		
Quadruped position arm and leg raise	2 sets, 30 sec hold	4 sets, 30 sec hold
Progression		
Prone on elbow	5 reps	10 reps
Half-moon pose	5 reps	10 reps
Stretching: hamstring stretch and erector spinae stretch	10 reps, 2 sets, 30 sec	15 reps, 4 sets, 30 sec
Cat and camel pose	10 reps	15 reps
Warrior pose	5 sets with 10 sec hold	10 sets with 10 sec hold
Reclining cobbler	5 sets	10 sets

Outcome measures

Pain

Pain intensity was assessed using the Numeric Pain Rating Scale (NPRS), scored from 0 to 10, where 0 indicated no pain and 10 represented the worst imaginable pain (r=0.96; validity=0.86-0.95) [21].

Disability

Functional impairment was evaluated using the Modified Oswestry Disability Index (MODI), a self-reported questionnaire of 10 questions reflecting the participants' functional activities. Each question has a score from 0 to 5, with a total score of 0-50. Scores 0-20 indicate minimal disability, 21-40 moderate disability, and 41-50 severe disability (r=0.89; validity=0.74-0.95) [22].

Strength

The Pressure Biofeedback Unit (PBU) was used with the participant in a hook-lying supine position. The three-chamber pressure cell was placed horizontally beneath the lumbar spine. The baseline pressure was set at 40 mmHg, and participants were instructed to maintain a neutral spinal position while keeping the pressure within 40±10 mmHg during the exercise [23]. 

Inclusion criteria

The study comprised male recreational runners aged 18-25 years who participated in running as a leisure activity rather than as professional athletes. Participants were required to train for a minimum of eight hours per week and have a history of NSLBP, coded in the electronic medical record as ICD-10 M54.5 (Low back pain, unspecified), for a minimum duration of three months. Individuals with sub-acute NSLBP and moderate pain intensity ranging from 5 to 7 on the NPRS were considered eligible. Additionally, only runners without any foot injuries and those willing to participate by providing written informed consent were included in the study.

Exclusion criteria

Individuals were excluded if they had a diagnosed specific pathological condition contributing to low back pain, including lumbar disc herniation/prolapse with radiculopathy, spondylolisthesis, spinal stenosis, vertebral fracture, spinal infection, spinal malignancy, or inflammatory spondyloarthropathy (e.g., ankylosing spondylitis), a recent musculoskeletal injury, or a history of recent surgical intervention involving the spine, upper extremity, or lower extremity. Participants currently enrolled in any structured physical training program or gym-based exercise regimen were also excluded. Further exclusion criteria included congenital deformities of the spine or extremities, neurological disorders affecting motor function, cauda equina syndrome, or any condition that could hinder participation in the intervention protocol.

Procedure

The investigation was carried out in a methodical and step-by-step manner. Before participant recruitment began, ethical clearance was acquired from the Institutional Ethics Committee, guaranteeing respect for ethical norms. All participants underwent screening to determine eligibility according to the specified inclusion and exclusion criteria. The goal and procedure of the study were described, and each eligible participant provided written informed consent. Demographic information was collected before baseline assessment. Pre-intervention measurements of pain, strength, and disability were recorded using the NPRS, PBU, and MODI, respectively. Following baseline assessment, participants diagnosed with LBP that was not specific were assigned at random to either the experimental group or the control group. Group A was given posterior chain resistance exercises along with spinal stabilization exercises, whereas Group B received spinal stabilization exercises alone. All exercises were demonstrated before the commencement of training. Each group experienced supervised training sessions three times for six weeks, once a week. Exercise intensity and volume were progressively increased after the third week. Following six weeks of intervention, all outcome measures were reassessed to determine changes from baseline. Baseline and post-intervention data were analyzed to assess the impact of the interventions.

Statistical analysis

Data analysis was conducted using relevant statistical techniques. Demographic and baseline characteristics were summarized using descriptive statistics. Using the paired t-test, within-group variations between pre-intervention and post-intervention scores for pain, strength, and disability were evaluated. The unpaired t-test was applied to contrast post-intervention outcomes within the two clusters. The relevance of the statistics was established at a p-value below the 0.05 threshold.

## Results

The results presented in Table 2 demonstrate a statistically significant improvement in all outcome measures following the intervention in Group A. The NPRS score decreased from 6.22±0.73 to 2.72±0.66 (t=18.89; p<0.0001), indicating a substantial reduction in pain intensity. The PBU score increased from 41.88±1.72 to 43.88±2.15 (t=8.74; p<0.0001), reflecting improved core muscle activation and lumbopelvic stability. Similarly, the MODI score decreased from 22.44±2.70 to 19.22±3.07 (t=11.24; p<0.0001), demonstrating a reduction in functional disability related to LBP. Since all p-values were less than 0.0001, the improvements observed in pain, core stability, and disability were considered extremely significant, indicating that the intervention was highly effective in enhancing clinical outcomes among participants.

**Table 2:** Comparison of pre- and post-intervention scores in Group A Data are presented as mean±standard deviation (SD). Within-group comparisons were performed using a paired t-test. Statistical significance was predefined at p<0.05.

Parameter	Pre (mean±SD)	Post (mean±SD)	T-value	P-value	Result
Numeric Pain Rating Scale (NPRS) score	6.22±0.73	2.72±0.66	18.89	<0.0001	Extremely significant
Pressure biofeedback unit (PBU) score	41.88±1.72	43.88±2.15	8.74	<0.0001	Extremely significant
Modified Oswestry Disability Index (MODI) score	22.44±2.70	19.22±3.07	11.24	<0.0001	Extremely significant

The results presented in Table 3 show a statistically significant improvement in all outcome measures following the intervention in Group B. The NPRS score decreased from 6.11±0.75 to 3.27±0.75 (t=15.296; p<0.0001), indicating a marked reduction in pain intensity. The PBU score increased from 41.83±2.01 to 42.94±2.18 (t=6.216; p<0.0001), suggesting improved core muscle activation and lumbopelvic stability. Likewise, the MODI score decreased from 22.00±2.35 to 18.88±2.49 (t=7.039; p<0.0001), reflecting a reduction in disability associated with NSLBP. As all p-values were less than 0.0001, the improvements in pain, core stability, and functional disability were considered extremely significant. These findings indicate that the intervention was effective in improving clinical outcomes among participants in Group B.

**Table 3:** Comparison of pre- and post-intervention scores in Group B Data are presented as mean±standard deviation (SD). Within-group comparisons were performed using a paired t-test. Statistical significance was predefined at p<0.05.

Parameter	Pre (mean±SD)	Post (mean±SD)	T-value	P-value	Result
Numeric Pain Rating Scale (NPRS) score	6.11±0.75	3.27±0.75	15.296	<0.0001	Extremely significant
Pressure biofeedback unit (PBU) score	41.83±2.01	42.94±2.18	6.216	<0.0001	Extremely significant
Modified Oswestry Disability Index (MODI) score	22.00±2.35	18.88±2.49	7.039	<0.0001	Extremely significant

Table 4 presents the between-group comparison of post-treatment NPRS scores between Group A and Group B using an independent t-test, which showed a statistically significant difference (p=0.025).

**Table 4:** Comparison of post-treatment Numeric Pain Rating Scale scores between Group A and Group B Data are presented as mean±standard deviation (SD). Within-group comparisons were performed using a paired t-test. Statistical significance was predefined at p<0.05.

Outcome	Group A (mean±SD)	Group B (mean±SD)	T-value	P-value	Interpretation
Numeric Pain Rating Scale (NPRS) score	2.72±0.66	3.27±0.75	2.342	0.025	Significant

The results presented in Table 5 indicate that the post-treatment PBU score was 43.88±2.15 in Group A and 42.94±2.18 in Group B. Although Group A demonstrated a slightly higher mean score, the independent (unpaired) t-test showed that the difference between the groups was not statistically significant (t=1.287; p=0.206). As the p-value exceeded the predefined significance level (p<0.05), both interventions produced comparable improvements in core stability, with no significant difference between the groups.

**Table 5:** Comparison of post-treatment pressure biofeedback unit scores between Group A and Group B Data are presented as mean±standard deviation (SD). Within-group comparisons were performed using a paired t-test. Statistical significance was predefined at p<0.05.

Outcome	Group A (mean±SD)	Group B (mean±SD)	T-value	P-value	Interpretation
Pressure biofeedback unit (PBU) score	43.88±2.15	42.94±2.18	1.287	0.206	Not significant

The results presented in Table 6 show that the post-treatment MODI score was 19.22±3.07 in Group A and 18.88±2.49 in Group B. The independent (unpaired) t-test revealed no statistically significant difference between the groups (t=0.356; p=0.723). Since the p-value was greater than the predefined significance level (p<0.05), both interventions were similarly effective in reducing disability, with neither treatment demonstrating superiority over the other.

**Table 6:** Comparison of post-treatment Modified Oswestry Disability Index scores between Group A and Group B Data are presented as mean±standard deviation (SD). Within-group comparisons were performed using a paired t-test. Statistical significance was predefined at p<0.05.

Outcome	Group A (mean±SD)	Group B (mean±SD)	T-value	P-value	Interpretation
Modified Oswestry Disability Index (MODI) score	19.22±3.07	18.88±2.49	0.356	0.723	Not significant

## Discussion

The present research investigated the effectiveness of combining posterior chain resistance exercises with spinal exercises for stability in recreational runners with NSLBP. The effectiveness of the combined exercise regimen was compared using activities for stabilizing the spine alone in improving pain, muscle strength, and disability. The results showed that both interventions resulted in notable reductions in pain, strength, and disability after six weeks of treatment. However, the combined intervention showed better results in terms of pain relief compared with spinal stabilization exercises alone.

In the present study, Group A showed a marked decrease in pain severity from baseline to post-intervention (p≤0.001). Group B, however, also displayed a notable improvement in pain reduction following six weeks of spinal stabilization training (p≤0.001). Inter-group analyses revealed that Group A had a statistically significant advantage (p=0.025), indicating that the addition of posterior chain resistance exercises produced greater pain reduction. The observed improvements may be attributed to enhanced strength and activation of the gluteal, hamstring, and lumbar extensor muscles, which play a vital role in maintaining lumbopelvic stability and facilitating efficient force transmission during running. Improved muscular support may reduce excessive loading on lumbar structures and enhance movement efficiency, thereby reducing pain. Similar findings were stated by França et al., who found that stabilization and strengthening exercises resulted in significant pain relief in participants with chronic LBP [3]. Similar findings were reported by Cairns et al., who demonstrated that spinal stabilization training was beneficial in mitigating symptoms and progressing functional outcomes in recurrent LBP [4].

Significant improvements in strength were observed in both groups after the completion of the intervention. Although Group A demonstrated a greater numerical improvement in strength than Group B, no statistically significant distinction was found between the groups (p=0.206). The improvements in strength observed in Group A could be linked to the progressive resistance training of the posterior chain musculature. The gains noted in Group B may be related to enhanced neuromuscular recruitment and stamina of trunk stabilizing muscles. These findings support the conclusions of Sannasi et al. [16], who emphasized the significance of both muscular strength and spinal stability in the treatment of LBP disorders. Furthermore, Goldby et al. reported that structured exercise programs incorporating strengthening and stabilization components can successfully enhance bodily function and muscular performance in people who have persistent low back discomfort [5].

There was statistical significance in both groups' improvements in disability outcomes following the intervention period (p≤0.001). Although Group A showed a higher decrease in disability scores, the disparity within the groups lacked statistical significance (p=0.723). This finding suggests that both therapies were equally successful in enhancing functional ability. Reduced pain levels, enhanced spinal stability, and improved muscular function may have contributed to better performance of daily and sporting activities. The current findings are in line with those of Saragiotto et al., who demonstrated significant reductions in disability following exercise therapies for motor control in individuals with chronic NSLBP [6]. Likewise, Hayden et al. found exercise therapy to be beneficial for improving function and lessening the impairment of individuals with chronic LBP [11].

The experimental group exhibited greater pain relief, which may be especially advantageous for recreational runners. Running places repetitive biomechanical demands on the lumbopelvic region, and weakness of the posterior chain musculature may contribute to abnormal force distribution and increased lumbar stress. Maselli et al. [19] found that low back discomfort was quite common among runners and emphasized the need to address biomechanical and muscular impairments during rehabilitation. The incorporation of posterior chain resistance exercises may therefore provide additional benefits by targeting important muscle groups in charge of force absorption, propulsion, and lumbopelvic control during running.

The current study's findings are supported by recent evidence highlighting the efficiency of resistance-based exercise programs in the management of LBP. According to Welch et al., resistance-based training interventions were efficient in improving physical function and reducing discomfort in individuals with chronic LBP [24]. Similarly, Steiger et al. reported that exercise programs focusing on strength as well as motor control were efficient in reducing disability and discomfort outcomes [25]. Gordon and Bloxham also concluded that exercise therapy, particularly programs involving strengthening exercises, continues to be a highly efficient conservative therapy for persistent LBP [26]. Aasa et al. reported that deadlift-based strengthening exercises considerably enhanced pain and functional outcomes in individuals with LBP. These findings support the inclusion of posterior chain resistance exercises in the present study [27].

Clinical implications

The investigation's findings show that both spinal stabilization exercises and posterior chain resistance exercises are successful methods of therapy for recreational runners with NSLBP. The inclusion of posterior chain resistance exercises could offer better pain relief and can therefore be incorporated into physiotherapy rehabilitation programs to optimize recovery, improve lumbopelvic function, and facilitate a safe return to running activities.

Strengths of the study

One of this study's main advantages was the randomized controlled design, which reduced the bias in allocation and enhanced the validity of the findings. The study specifically focused on recreational runners, a group of people who have gotten little attention in previous LBP research. Additionally, validated outcome measures were employed to evaluate pain, strength, and disability, allowing the comprehensive assessment of treatment effectiveness.

Limitations of the study

Only men were involved in the study. Participants were 18-25 years of age, which restricts the ability to generalize the findings to female runners and different age groups. The sample size was insufficient, and the intervention period was restricted to six weeks. No extended surveillance was done, making it challenging to evaluate the feasibility of the noted advancements.

Future scope

Larger and more varied groups should be included in future research, including female recreational runners and athletes from different age groups. Long-term monitoring assessments are recommended to evaluate the persistence of medical care effects. Additional research could possibly look into the influence of posterior chain resistance exercises on running biomechanics, athletic performance, recurrence rates of LBP, and sport-specific functional outcomes.

## Conclusions

This investigation found that both posterior chain resistance exercises combined with spinal stabilization exercises and spinal stabilization exercises alone were effective in lessening pain, improving strength, and decreasing disability among recreational runners with NSLBP. After six weeks, both groups showed notable improvements from the intervention. However, the combination of posterior chain resistance exercises with spinal stabilization exercises resulted in considerably more pain relief in comparison to spinal stabilization exercises alone. No notable between-group disparities were discovered for strength and disability outcomes. Therefore, incorporating posterior chain resistance exercises in recovery programs could provide additional benefits for pain management and enhance overall recovery in recreational runners with NSLBP.
